# Survival and associated factors among patients with oral squamous cell carcinoma (OSCC) in Mulago hospital, Kampala, Uganda

**DOI:** 10.1186/s41199-018-0036-6

**Published:** 2018-10-26

**Authors:** Juliet Asio, Adriane Kamulegeya, Cecily Banura

**Affiliations:** 1 HIV Reference Laboratory, Uganda Virus Research Institute, P. O. Box 49, Entebbe, Uganda; 2 Department of Dentistry, College of Health Sciences, Makerere University, P. O. Box 6717, Kampala, Uganda; 3 Child Health and Development Centre, College of Health Sciences, Makerere University, P. O. Box 6717, Kampala, Uganda

**Keywords:** Oral squamous cell carcinoma, Uganda, Survival, Clinical-pathological presentation

## Abstract

**Background:**

Despite improvements in diagnosis and patient management, survival and prognostic factors of patients with oral squamous cell carcinoma (OSCC) remains largely unknown in most of Sub Saharan Africa.

**Objective:**

To establish survival and associated factors among patients with oral squamous cell carcinoma treated at Mulago Hospital Complex, Kampala.

**Methods:**

We conducted a retrospective cohort study among histologically confirmed oral squamous cell carcinoma (OSCC) patients seen at our centre from January 1st 2002 to December 31st 2011. Survival was analysed using Kaplan-Meier method and comparison between associated variables made using Log rank-test. Cox proportional hazards model was used to determine independent predictors of survival. *P*-values of less than 0.05 were considered statistically significant.

**Results:**

A total of 384 patients (229 males and 155 females) were included in this analysis. The overall mean age was 55.2 (SD 4.1) years. The 384 patients studied contributed a total of 399.17 person-years of follow-up. 111 deaths were observed, giving an overall death rate of 27.81 per 100 person-years [95% CI; 22.97–32.65]. The two-year and five-year survival rates were 43.6% (135/384) and 20.7% (50/384), respectively. Tumours arising from the lip had the best five-year survival rate (100%), while tumours arising from the floor of the mouth, alveolus and the gingiva had the worst prognosis with five-year survival rates of 0%, 0% and 15.9%, respectively. Independent predictors of survival were clinical stage (*p* = 0.001), poorly differentiated histo-pathological grade (*p* <  0.001), male gender (p = 0.001), age > 55 years at time of diagnosis (*p* = 0.02) and moderately differentiated histo-pathological grade (*p* = 0.027). However, tobacco & alcohol consumption, tumour location and treatment group were not associated with survival (*p* > 0.05).

**Conclusions:**

The five-year survival rate of OSCC was poor at 20.7%. Male gender, late clinical stage at presentation, poor histo-pathological types and advanced age were independent prognostic factors of survival. Early detection through screening and prompt treatment could improve survival.

## Background

Oral squamous cell carcinoma (OSCC) is a potentially disfiguring and debilitating disease that affects the physical appearance of patients and devastates their self-esteem. Globally, over 175,000 cases are diagnosed annually [1]. The age-adjusted incidence and mortality rates of OSCC increases with age and are greater in males than females [2]. It is well established that tobacco use and alcohol consumption are significant risk factors [3]. Some studies suggest that among people living with HIV, the risk of oral cancer is elevated [4].

The risk factors for Human Papilloma Virus (HPV) positive OSCC are mainly related to sexual habits rather than to tobacco and alcohol use in HPV negative OSCC [5]. Furthermore, over the past decade, oncogenic HPV type 16 has been linked to the development of some oral pharyngeal cancers but the association with oral cancer proper was not evident [6]. The detection of HPV DNA in some oral pharyngeal cancers has been linked to a favourable prognosis particularly among males [7]. Sub Saharan Africa (SSA) having a high burden of infection related cancers may provide unique circumstances in oral cancers worth researching.

Despite improvements in diagnostic facilities and patient management, survival and prognostic factors of OSCC remain unknown in most of SSA. Data from the Kampala Cancer Registry showed that oral cancer (ICD-10 C00-C06) was a rare disease that contributed 1.1% cases in Uganda [8]. However, there is paucity of data on survival and prognostic factors of oral cancers in Uganda. Therefore, the purpose of this study was to establish survival rates and determine independent prognostic factors of survival among patients with OSCC.

## Methods

### Study design and setting

Records of patients with histologically confirmed OSCC seen at Mulago Hospital Complex from January 1^st^ 2002 to December 31^st^ 2011 were reviewed.

Mulago hospital is a national referral hospital, which has the only functional oral and maxillofacial surgery unit and the only radiotherapy unit serving the whole of Uganda and the neighbouring countries. Additionally, Mulago Hospital Complex shares location with the Uganda Cancer Institute (UCI) that provides chemotherapy treatment and care of cancer patients in Uganda and neighbouring countries. Records of patients with OSCC were retrieved from the Oral and Maxillofacial department and their socio-demographic, clinical and pathological data was abstracted. At both UCI and the Radiotherapy department, registers were used to identify patients with OSCC. Records of patients with OSCC were then retrieved and their details recorded.

### Study population

The sample size was determined using the following assumptions: the log rank comparisons of the probability of experiencing death in 5 years between patients with early disease and those with advanced disease at 0.47, power of 80%, 5% significance level, an effect size of 1.595 and adjusting for loss to follow-up of 10%. The total number of (events) deaths that were required was 149 and at least 270 participants were required for this study.

Consecutive records of 384 index patients with a histological diagnosis of OSCC seen at Mulago Hospital Complex were retrieved for assessment. Records with missing important variables (e.g. date of diagnosis, site of lesion) or those with vague histological diagnosis (such as ‘moderately-well’ differentiated, ‘poorly-well’ differentiated), those of patients who presented with second primaries and patients who were referred to Hospice Uganda for terminal care, were excluded from the study. To eliminate duplicate recruits, patient demographic characteristics at different entry points of care were compared using hospital identification numbers and patient details. From each eligible record, demographic characteristics, pre-operative tumour characteristics, TNM stage, tobacco and alcohol usage, treatment instituted, length of follow-up and survival status were abstracted. To determine the nodal status in TNM staging, both clinical and radiological findings were assessed whenever available, while the evaluation of metastases was based on chest x-ray reports. In some cases, follow-up phone calls were made to patients or their next of kin with recorded telephone contacts in order to ascertain the status of the patient.

### Statistics and analysis

Statistical analyses were performed using STATA Version 12. The length of follow-up was defined as the period in months between the date of histological diagnosis and time to death or censoring. Cases were classified as alive, dead (if date of death was recorded) or lost to follow-up (date of last visit as recorded in patient’s file). Baseline characteristics for the patients were described using percentages for categorical variables and medians for continuous variables.

Survival was calculated using the Kaplan-Meier analysis and the significance of the difference between survival curves for each variable was determined using the Breslow-test. *P*-values less than 0.05 were considered statistically significant. The Cox proportional hazards model was used to obtain independent predictors of survival. Construction of the final model was done in stages. Initially, all variables with a *p* value < 0.25 at univariate analysis were included in the multivariable model. To test for goodness of fit of the multivariable model a plot of Nelson–Aalen cumulative hazard estimate against Cox Snell residuals was plotted.

## Results

Records of 512 patients were retrieved. One hundred twenty eight (25.0%) records were excluded due to missing data including: vague or no histological diagnosis, patients with second primaries, and patients referred to Hospice Uganda for terminal care. Therefore, 384 (75.0%) records were included in the analysis. In addition, 70 (13.7%) records with no data on clinical stage at presentation were excluded from survival analysis.

### Socio-demographic characteristics, alcohol consumption and tobacco use

The mean age of the 384 patients included in this study was 55.2 years with a standard deviation of 4.1 years. There were 229 (59.6%) males and 155 (40.4%) females. Males had a mean age of 55.8 years (SD = 19.9 years), whereas females had a mean age of 55.6 years (SD = 15.9 years). Most patients were in their sixth decade 104 (27.1%). Most patients came from the western region of the country 130 (33.9%). Of the 214 patients with a history of education background, less than 40% had attained secondary level education (Table 1). Compared to females, more males reported use of tobacco and alcohol.

**Table 1:** Demographic, clinical and pathological characteristics of 384 OSCC patients

Characteristic	n(%)
Gender	
Male	229(59.6)
Female	155(40.4)
Age (years)	
Mean (SD)	55.2(4.1)
Tobacco use	
User	147(54.7)
Non-User	122(45.4)
Alcohol use	
User	140(52.6)
Non-User	126(47.4)
Geographical region	
Central	132(34.4)
Eastern	72(18.7)
Northern	41(10.7)
Western	109(28.4)
Non-Ugandan	30(7.8)
Education level	
Tertiary	27(12.6)
Secondary	44(20.6)
Primary	68(31.8)
None	75(35.0)
Histo-pathological grade	
Well differentiated	198(51.5)
Moderately differentiated	102(26.6)
Poorly differentiated	84(21.9)
Treatment modality	
Surgery	38(9.9)
Radiotherapy	224(58.3)
Chemotherapy	4(1.0)
Surgery + Radiotherapy	41(10.7)
Surgery + Chemotherapy	8(2.1)
Surgery + Radiotherapy +Chemotherapy	8(2.1)
Radiotherapy + Chemotherapy	3(0.8)
None	58(15.1)

### Sub-site tumour presentation, histopathological grading and clinical stage

The distribution of primary tumour sites, spread and clinical stage of OSCC is presented in Table 2. In descending order, the tongue (34.1%), palate (13.5%), buccal mucosa (13.3%) and floor of the mouth (12.2%) were the commonest primary sites.

**Table 2:** Sub-site distribution, TNM classification and clinical stage at presentation of 384 patients with OSCC

Variable	Number	Percentage
Site		
Alveolus	18	4.7
Buccal Mucosa	51	13.3
Floor of mouth	47	12.2
Gingiva	43	11.2
Lip	16	4.2
Palate	52	13.5
Tongue	131	34.1
Other^α^	26	6.8
T (Tumour)		
1	41	10.7
2	142	37.0
3	91	23.7
4	62	16.1
X	48	12.5
N (Nodal involvement)		
0	151	39.3
1	63	16.5
2	106	27.6
3	22	5.7
X	42	10.9
M (Metastasis)		
0	228	59.4
1	86	22.4
X	70	18.2
Clinical Stage (Based on TNM staging system)		
I	23	6.0
II	57	14.8
III	148	38.5
IV	86	22.5
X	70	18.2

^α^Other includes Commissure, Buccal sulcus, Retromolar trigone, Sublingual salivary glands

X Missing data

Majority 51.6% (*n* = 198) of patients had well differentiated tumours, and about one-fifth (21.9%, *n* = 84) had poorly differentiated tumours. Majority (61%) of the identified OSCC were in TNM stage III and IV (Table 1).

### Survival pattern of 384 patients with OSCC

The 384 patients studied contributed a total of 399.17 person–years of follow-up. One hundred eleven deaths were observed, giving an overall death rate of 27.81 per 100 person–years [95% CI; 22.97–32.65]. The overall average survival time for patients with OSCC was 375 days. The two-year and five-year survival rates were respectively 43.6% (135/384) and 20.7% (50/384), (Table 3).

**Table 3:** Survival Pattern of 384 patients with OSCC

Time (years)	Total number	Deaths	Censored	Survival	95% Confidence Interval
0	384	50	199	0.824	0.775 0.864
1	135	40	45	0.531	0.450 0.606
2	50	8	11	0.436	0.347 0.521
3	31	10	6	0.280	0.189 0.378
4	15	0	2	0.280	0.189 0.378
5	13	3	3	0.207	0.117 0.314

**Fig. 1:**
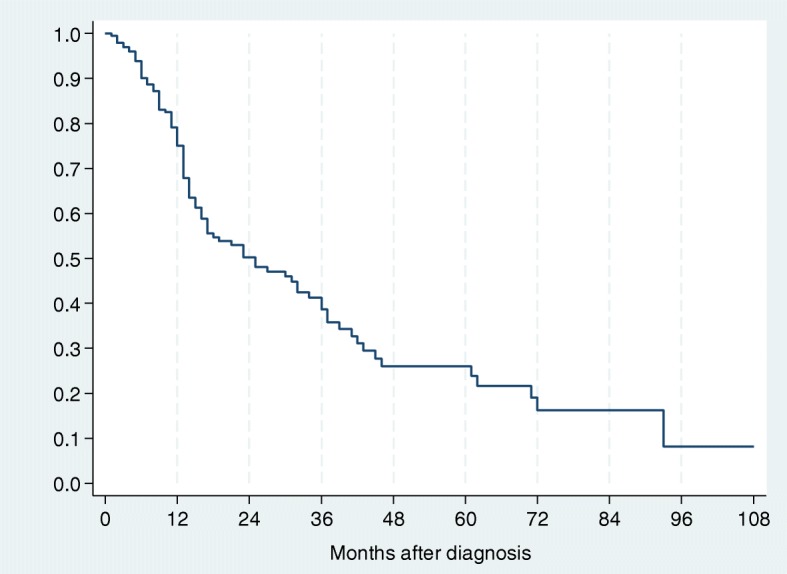
Kaplan–Meier estimates for 384 patients with OSCC

**Fig. 2:**
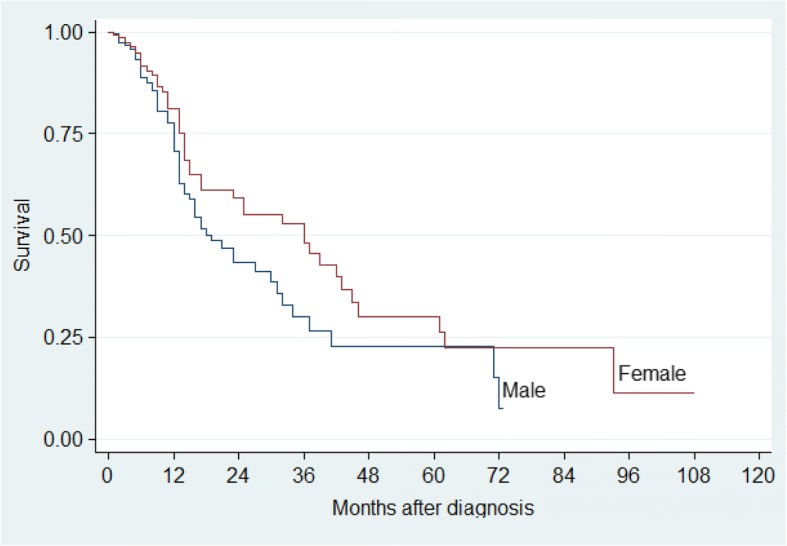
Kaplan–Meier estimates by Gender for patients with OSCC

**Fig. 3:**
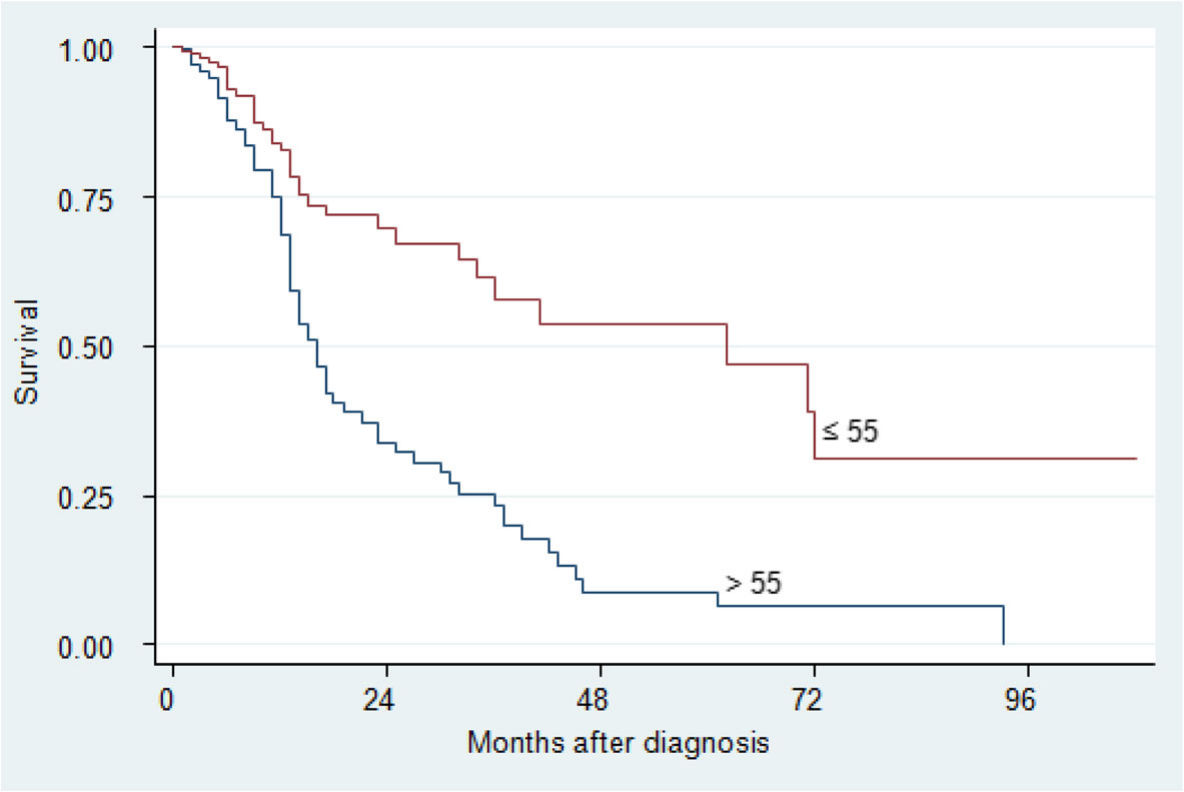
Kaplan–Meier survival estimates by Age for patients with OSCC

**Fig. 4:**
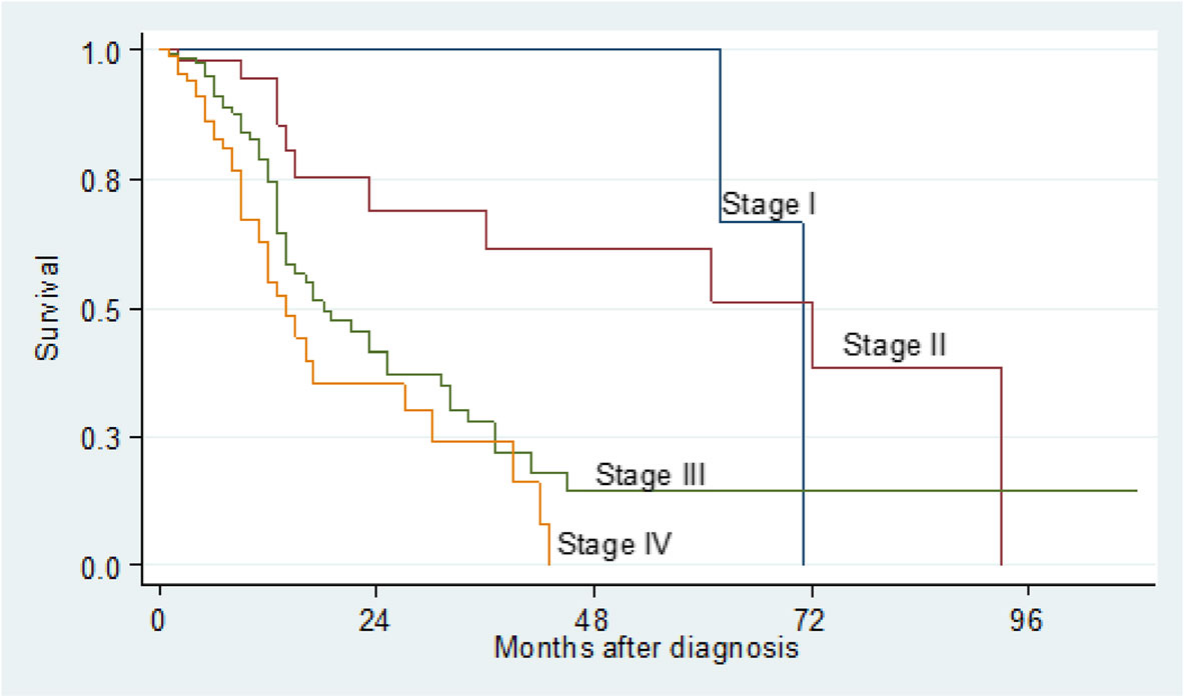
Kaplan–Meier survival estimates by Clinical Stage for patients with OSCC

**Fig. 5:**
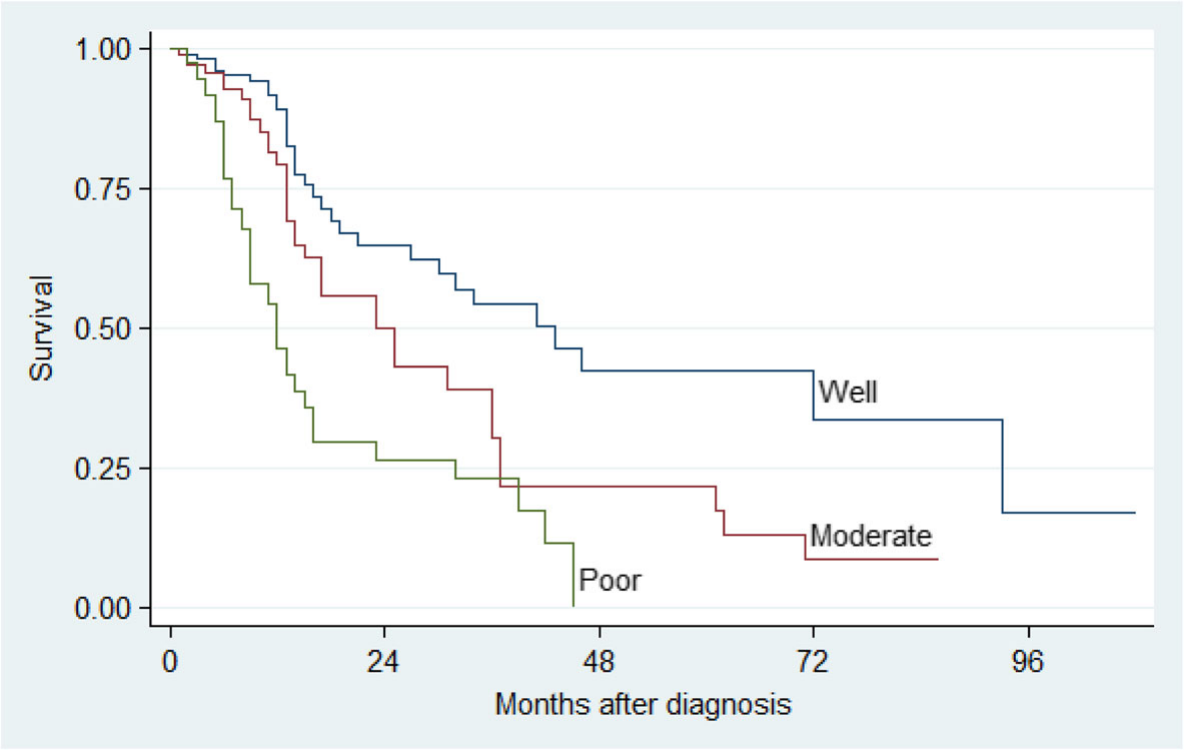
Kaplan–Meier survival estimates by Histo-pathological Grade for patients with OSCC

The two-year and five-year survival rates were significant for age (*p* = 0.001), clinical stage (*p* <  0.001) and pathological stage (*p* <  0.001). There was no difference in gender, tumour localisation, treatment group and in patients with or without a history of either tobacco or alcohol consumption (*p* > 0.05), (Table 4). Kaplan–Meier analysis and log-rank test were used for bivariate analysis. Kaplan–Meier curves were constructed for all patients and for significant variables (Figs. 1, 2, 3, 4 and 5).

**Table 4:** Univariate Analysis of 384 Patients with OSCC

Variable	Survival rate (%)		*P* value (log-rank)
	2-year	5-year	
Gender			
Male	43.3	22.9	0.053
Female	59.2	30.1	
Age (years)			
≤ 55	69.7	53.7	0.001
> 55	34.0	8.8	
Tobacco Use			
User	47.7	15.8	0.091
Non-User	49.6	29.8	
Alcohol Use			
User	46.6	26.3	0.460
Non-User	50.7	21.0	
Tumour Location			
Alveolus	59.3	0.0	0.255
Buccal mucosa	47.0	19.6	
Floor of mouth	34.5	0.0	
Gingiva	39.7	15.9	
Lip	100.0	100.0	
Palate	52.3	43.6	
Tongue	53.3	21.2	
Other	53.9	53.9	
Clinical Stage			
I	100.0	100.0	< 0.001
II	69.1	61.5	
III	41.7	14.5	
IV	35.4	0.0	
Histo-pathological grade			
Well differentiated	64.9	42.2	< 0.001
Moderately differentiated	50.1	21.7	
Poorly differentiated	26.4	0.0	
Treatment Group			
Surgery	73.8	61.5	0.103
Radiotherapy	47.5	27.6	
Chemotherapy	100.0	0.0	
At least 2	55.3	12.3	

Other – Commissure, Buccal sulcus, Retromolar trigone, Sublingual salivary glands

At least 2 – Surgery and Radiotherapy or Surgery and Chemotherapy

*P* value is for 5-year survival

### Predictors of survival among OSCC patients

Construction of the final model containing variables found to be independently associated with survival of oral cancer was made using Cox proportional hazards model. A model which included all variables that had a *P*–value of less than 0.25 at univariate analysis was formed (Table 5). These included clinical stage, pathological variant, treatment group, gender, age and tobacco use. The variable tumour site (*p* = 0.26) was included on the basis of previous studies.

**Table 5:** Model showing the combined effect of significant variables

Variables	Hazard Ratio	95% Confidence Interval	*P* value	
Clinical stage				
I & II	1			
III & IV	2.998	1.584	5.674	0.001
Histo-pathological grade				
Well differentiated	1			
Moderately differentiated	1.756	1.065	2.897	0.027
Poorly differentiated	2.985	1.798	4.957	< 0.001
Age				
≤ 55	1			
> 55	1.022	1.008	1.036	0.002
Gender				
Male	1			
Female	0.482	0.310	0.749	0.001

The model was tested to verify whether the assumption of proportionality between early-stage and late stage disease patient categories. The proportionality of hazards assumption of the model was tested as a whole, and for each variable using the global test and the extended Cox model. The model was not significant based on the Schoenfeld’s test (*p* = 0.838) and the extended Cox model indicating that the data did not violate the proportional hazards assumption. There model was tested for interaction and confounding, using clinical stage as the main predictor of survival. The final model was thus determined as:

$$ \mathbf{h}\left(\mathbf{t},\mathbf{x}\right)={\mathbf{h}}_{\mathbf{0}}\ \mathbf{\exp}\ \left(\mathbf{1.09}\mathbf{8clinical}\ \mathbf{stage}\left(\mathbf{III}\&\mathbf{IV}\right)+\mathbf{0.02}\mathbf{7moderately}\ \mathbf{differentiated}\ \mathbf{tumour}+\mathbf{01.09}\mathbf{4poorly}\ \mathbf{differentiated}\ \mathbf{tumour}+\mathbf{0.02}\mathbf{3age}-\mathbf{0.73}\mathbf{1female}\right) $$

The model itself was significant (*p* <  0.001). It was also tested for goodness of fit using a plot of Nelson–Aalen cumulative hazard estimate against Cox Snell residuals which gave a good model.

### Assessment of selection bias on participants lost to follow-up

A total of 141 (44.9%) participants were lost to follow-up during the study. This rate is higher than the acceptable 15%. The characteristics of these patients were assessed to determine the possibility of selection bias. The patients who were lost to follow-up had similar characteristics to those who remained in the study except with reference to treatment group, as shown in Table 6 below.

**Table 6:** Comparison of characteristics of patients enrolled and those lost to follow-up

Variable	Alive/Dead		Lost to follow-up		*P* value
	Total (*n* = 173)	%	Total (*n* = 141)	%	
Gender					
Male	105	60.7	81	57.4	0.560
Female	68	39.3	60	42.6	
Age (years)					
Median (IQR)	56	(44.5–66)	60	(47.5–66)	0.284
Tobacco use					
User	79	51.6	68	58.6	0.254
Non-user	74	48.4	48	41.4	
Alcohol use					
User	85	55.9	55	48.2	0.215
Non-user	67	44.1	59	51.8	
Tumour location					
Alveolus	6	3.6	10	7.1	0.764
Buccal mucosa	17	9.8	18	12.8	
Floor of mouth	22	12.7	13	9.2	
Gingiva	23	13.3	15	10.6	
Lip	7	4.0	9	6.4	
Palate	24	13.9	21	14.9	
Tongue	62	35.8	47	33.3	
Other	12	6.9	8	5.7	
Clinical stage					
I	16	9.2	7	5.0	0.410
II	31	17.9	26	18.4	
III	79	45.7	69	48.9	
IV	47	27.2	39	27.7	
Histo-pathological grade					
Well differentiated	80	45.8	89	63.1	9.351
Moderately differentiated	46	26.8	32	22.7	
Poorly differentiated	47	27.4	20	14.2	
Treatment group					
Surgery	18	11.8	17	15.0	0.057
Radiotherapy	110	71.9	72	63.7	
Chemotherapy	2	1.3	1	0.9	
At least 2	23	15	23	20.4	

## Discussion

This study, to the best of our knowledge presents one of a few on survival of OSCC patients in Sub Saharan Africa. It showed poor survival of patients with OSCC (20.7%) after five years and almost half of them (43.6%) had died within 2 years of diagnosis. Our findings are similar to the low five-year survival rate observed in Egypt for intra oral cancers (20.8%) [9]. However, better survival especially for stages III and IV has been reported in resource rich countries like Taiwan 26.6% and 11.8% [10], Brazil 32.6% and 24.5% [11] and the USA [12]. This discrepancy may be a reflection of better screening programs for early detection of cases and better treatment modalities, which ultimately improves survival, in the better resourced countries. The case for standardised treatment and its effect on survival irrespective of the difference in ethnicity and economic status has already been made for all head and neck squamous cell carcinomas [13].

Gender had a significant effect on survival in our study with the risk of death two times greater in males compared to females (Table 5 and Fig. 2). The effect of gender on survival remains mixed and unclear. Whereas some studies suggest a greater survival for females [14, 15], Mehta et al. reported lesser improvement in survival for females with oral cavity and oral pharyngeal carcinomas [16]. Other studies have reported no significant difference in survival between males and females [10]. It is believed that more males than females are affected by OSCC and have worse survival because of their increased exposure to tobacco and alcohol [2]. Furthermore, the males have poor health seeking behaviours, which may translate into delayed diagnosis and treatment initiation [17].

Age was a significant prognostic factor for survival in this study (Table 5 and Fig. 3). Patients who presented with OSCC and were above 55 years had a significantly shorter survival time as compared to those who were younger (*p* = 0.001). Our findings are consistent with studies conducted in Brazil [11], USA [16], Taiwan [10] and Egypt [9]. There seems to be a general agreement that the lower survival among older patients may be related to the higher rates of co-morbidities associated with ageing. It is also possible that these co-morbidities preclude the older patients from long surgical interventions which disadvantages their survival yet radiotherapy alone has been reported to lead to worse prognosis [11, 18]. In addition, with the emerging role of HPV, in oral and oral pharyngeal cancers, it may be that the younger population has a different causative factor hence better outcomes. However, a study from Mbarara in western Uganda showed a low prevalence of HPV among the head and neck cancers [19].

Education level, alcohol consumption and tobacco smoking were not significant predictors of survival. However, determination of cigarette smoking and tobacco and alcohol use, post event, may not be accurate thus making determination of their influence on patient survival hard to establish [20]. Education level is a surrogate for socio-economic status which has been shown to affect survival. Therefore, more research needs to be done to establish why it had no effect in our study.

Tumour site was not an independent predictor of survival. This was consistent with other studies [21] but different from others [9, 11, 14]. The possibility of misclassification of original OSCC site is high given the complex anatomy of the oral cavity coupled with delayed presentation seen among our patients [22]. In advanced stages, there could be an overlap of oral tumours that arise from adjacent structures leading to misclassification. In this study, about two-thirds of patients presented with late stage disease making misclassification of the original site of OSCC highly likely.

OSCC arising from the lip had the best five-year survival rate (100%) consistent with results from other studies [9, 14]. This may be because lip cancer is noticed earlier by patients and so they tend to seek care earlier. On the other hand, the floor of the mouth, alveolus and the gingiva had the worst five-year survival rates of 0%, 0% and 15.9%, respectively. Our results are different from those obtained from other studies which showed that the tongue had the lowest survival rate [11, 12]. The differences in survival by tumour site could arise from the ease of early diagnosis, accessibility for excision of the tumour with sufficient surgical margin and the different lymph node involvement that each site presents. However, given the previously reported late presentation among our patients [22], tongue carcinomas may progress into the floor of the mouth making it hard to know the original site. In addition, some anatomic sites manifest greater metastatic capacity due to high lymphatic drainage [17].

We found an inverse relationship between tumour stage and survival (*p* <  0.001), which was consistent with other studies [9, 11, 18, 21, 23]. The five-year survival rates were 100%, 61.5%, 14.5% and 0% for patients with stages I, II, III and IV, respectively. A study conducted in Egypt found similar survival rates of 100%, 65.5%, 42.2% and 0% for stages I, II, III and IV disease, respectively [9]. However, the rates in our study are much lower than those reported by two studies that investigated the outcomes of OSCC after surgical and/or radiation therapy in America [24] and Taiwan [21]. The much lower survival rates reported in this study could be a reflection of the study population that comprised more of patients in clinical stages III and IV than those in stages I and II at presentation, which was much higher than those reported by other studies.

Histo-pathological grading was a significant predictor of survival in this study. It is widely reported that prognosis is better with early stage well differentiated disease than other histo-pathological types [21]. In fact, the risk of death increased with less well differentiated tumours in this study. Patients with poorly and moderately differentiated tumours had three fold and almost two fold risk of death, respectively, compared to those who had well differentiated tumours. However, it is worth noting that some reports have not shown tumour grade to have an effect on survival [11, 23].

The type of treatment received by the patient was not a predictor of survival in this study. Of the 384 patients, about two-thirds (67.3%) received at least one form of treatment (Table 3). Radiotherapy, either alone or in combination with surgery was the most common treatment modality. Patients treated with surgery showed the highest two-year and five-year survival rates followed by surgery and radiotherapy. However, most untreated patients died within 5 years and so did many of the patients treated with radiotherapy alone or chemotherapy alone. However, several studies where surgery was the primary mode of treatment found treatment modality as a significant predictor of survival [10–12, 21]. The treatment modality is dependent on stage of disease and other parameters such as anatomical site, tumour size, distant metastasis, histological type and lymph node involvement [12]. While surgery alone may be recommended for patients with early stage disease, adjuvant radiotherapy or chemotherapy is indicated for patients with advanced stages [12].

The large number of patients lost to follow up could also explain why treatment modality was not a significant predictor of survival since patients who were lost to follow-up had a borderline difference (*p* = 0.057) from those who were not, with respect to treatment group. Patients who were lost to follow-up were most likely those who were assigned to treatment modalities that required repeated visits such as chemotherapy and radiotherapy for advanced stage disease. It is also possible that many of the patients lost to follow-up were travelling long distances to access these treatment modalities, which would make re-visits expensive. Furthermore, patients were classified solely on their treatment status without taking into consideration the dosage, duration and compliance with treatment received. In our setting sometimes surgery is not an option due limited surgical space. Sometimes this may lead to significant delays in accessing the service thus disease progression and change in stage [25]. This does have a significant effect on outcomes. It is not any different when it comes to radiotherapy were machine breakdowns and patient load likewise lead to delayed treatment compromising outcomes [26].

### Limitations

Our study was a hospital and not population-based study. It may therefore not be a representative sample of all the OSCC in Uganda. Data on HIV status of the patients and detection of HPV DNA in the tumours was not available. Seventy (13.7%) records with no data on clinical stage at presentation were excluded from survival analysis. However, this did not affect the power of the study given that we sampled 384 records, compared to 270 required for this study.

## Conclusion

Poor survival rates of oral cancer were recorded in this study, with two-year and five-year survival rates at 43.6% and 20.7% respectively. Male gender, late clinical stage at presentation due to delay in seeking medical care, poor histo-pathological types and advanced age were independent predictors of survival. Early detection through screening and prompt treatment could improve survival.

## Abbreviations

CI: Confidence Interval
CMV: Cytomegalovirus
EBV: Epstein barr virus
HIV: Human immunodeficiency virus
HPV: Human Papilloma Virus
HR: Hazard Ratio
HSSP: Health sector strategic plan
IQR: Inter-quartile range
LTC: Lymphoma treatment centre
MoH: Ministry of Health
NCD: Non-communicable disease
OSCC: Oral Squamous Cell Carcinoma
SD: Standard deviation
SSA: Sub-Saharan Africa
STC: Solid tumour treatment centre
TNM: Tumour node metastasis
UCI: Uganda Cancer Institute
WHO: World Health Organisation
